# Acral Exogenous Cutaneous Pigmentation Caused by Contact with a Darkling Beetle (Coleoptera: Tenebrionidae) in a Young Patient in the state of Amazonas

**DOI:** 10.1590/0037-8682-0298-2024

**Published:** 2025-01-27

**Authors:** Iara de Melo Freire, Carolina Souza de Oliveira, Marcella Caldeira Camisasca Souza, Virgínia Vilasboas Figueiras, Luciana Mendes dos Santos

**Affiliations:** 1 Fundação de Medicina Tropical Heitor Vieira Dourado, Departamento de Dermatologia, Manaus, AM, Brasil.; 2 Hospital Universitário Getúlio Vargas, Departamento de Dermatologia, Manaus, AM, Brasil.

**FIGURE 1:**
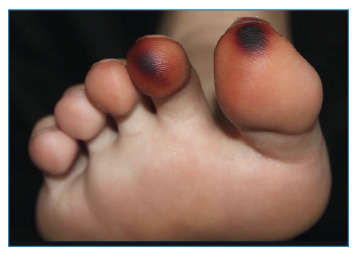
Blackened macules affecting the 1st and 2nd toes of the right foot.

**FIGURE 2:**
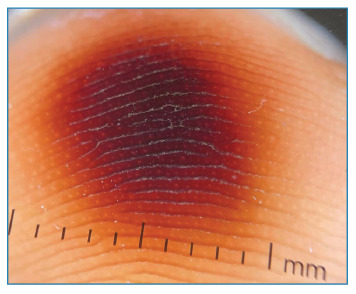
Dermoscopy showing brownish exogenous pigment in a parallel ridge pattern.

A 27-year-old male patient from Manaus, the capital of the Amazonas state, sought medical attention after reporting direct contact with a beetle found in his shoe. Approximately 2 hours after wearing the shoe, the patient experienced burning sensation and paresthesia in the toes of his right foot, without presenting any systemic complaints. Physical examination revealed blackened oval macules surrounded by an erythematous halo, predominantly affecting the first and second toes of the right foot (Figure 1). Dermoscopic assessment showed a brown pigmentation pattern along the ridge (Figure 2). The patient was admitted for observation and underwent laboratory tests, which yielded normal results. After 3 days, the lesions showed gradual improvement, and the patient was discharged. 

**FIGURE 3:**
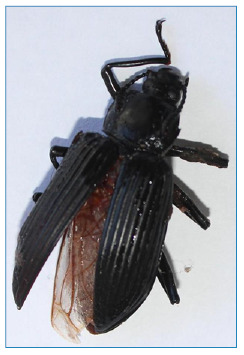
Coleoptera Tenebrionidae (darkling beetle).

The beetle involved belongs to the order Coleoptera and family Tenebrionidae, which are known for their widespread global distribution, predominance in tropical and temperate regions (Figure 3). Although the family Tenebrionidae is not commonly associated with bites or dermatitis in humans, occasional reports indicate the possibility of cutaneous reactions due to contact with these insects. These beetles produce hemolymph containing various chemical substances such as hydrocarbons and quinones, which can cause significant pigmentation when in contact with human skin^1^ ^,^ ^2^. As a defense mechanism, they release dark-red chemical substances when threatened^3^. Conservative treatment with vigilant observation is the preferred treatment approach. This report describes a rare case of exogenous cutaneous pigmentation caused by direct contact with a beetle from the family Tenebrionidae, which resolved spontaneously.
